# mRNA N^6^-methyladenosine methylation of postnatal liver development in pig

**DOI:** 10.1371/journal.pone.0173421

**Published:** 2017-03-07

**Authors:** Shen He, Hong Wang, Rui Liu, Mengnan He, Tiandong Che, Long Jin, Lamei Deng, Shilin Tian, Yan Li, Hongfeng Lu, Xuewei Li, Zhi Jiang, Diyan Li, Mingzhou Li

**Affiliations:** 1 Institute of Animal Genetics and Breeding, College of Animal Science and Technology, Sichuan Agricultural University, Chengdu, Sichuan, China; 2 Novogene Bioinformatics Institute, Beijing, China; Huazhong University of Science and Technology, CHINA

## Abstract

N^6^-methyladenosine (m^6^A) is a ubiquitous reversible epigenetic RNA modification that plays an important role in the regulation of post-transcriptional protein coding gene expression. Liver is a vital organ and plays a major role in metabolism with numerous functions. Information concerning the dynamic patterns of mRNA m^6^A methylation during postnatal development of liver has been long overdue and elucidation of this information will benefit for further deciphering a multitude of functional outcomes of mRNA m^6^A methylation. Here, we profile transcriptome-wide m^6^A in porcine liver at three developmental stages: newborn (0 day), suckling (21 days) and adult (2 years). About 33% of transcribed genes were modified by m^6^A, with 1.33 to 1.42 m^6^A peaks per modified gene. m^6^A was distributed predominantly around stop codons. The consensus motif sequence RRm^6^ACH was observed in 78.90% of m^6^A peaks. A negative correlation (average Pearson’s *r* = -0.45, *P* < 10^−16^) was found between levels of m^6^A methylation and gene expression. Functional enrichment analysis of genes consistently modified by m^6^A methylation at all three stages showed genes relevant to important functions, including regulation of growth and development, regulation of metabolic processes and protein catabolic processes. Genes with higher m^6^A methylation and lower expression levels at any particular stage were associated with the biological processes required for or unique to that stage. We suggest that differential m^6^A methylation may be important for the regulation of nutrient metabolism in porcine liver.

## Introduction

Over 100 types of chemical modification to RNA have been described [1], most of which are formed by specific enzymatic modification of the primary RNA transcript during the tRNA complex maturation process [2, 3]. N^6^-methyladenosine (m^6^A) is one of the most prevalent modifications of eukaryotic mRNAs [4] with conserved topology across yeast [5], *Arabidopsis thaliana* [6, 7], *Drosophila* [8], mouse and human [9, 10]. Most of the m^6^A sites share a similar consensus m^6^A motif, RRm^6^ACH, where R represents a purine and H represents a non-guanine base [9]. It has been estimated that over one-third of genes in mouse and human transcriptomes are m^6^A methylated [9], and this figure rises to over 70% in *Arabidopsis* [7]. Transcriptome-wide analysis of m^6^A in mouse and human shows m^6^A sites preferentially appearing at distinct landmarks, around stop codons and within long internal exons [9, 10]. In *Arabidopsis*, m^6^A sites are also located immediately following transcription start sites (TSS) [6], which is thought to be the main difference between m^6^A patterns in plants and animals.

Recent evidences show that m^6^A methylation is involved in vast aspects of RNA metabolism in mammals [11–13], and has diverse characteristics in cells (HEK293T and embryonic stem cells) and tissues (brain and liver) [9, 10, 14]. Genes with m^6^A methylation mainly enriched in biologically important pathways, such as regulation of gene expression, differentiation and metabolism [6, 7, 9]. Nonetheless, m^6^A methylation profiles and functions in tissue at postnatal developmental stages have rarely been investigated.

The liver is a vital organ) with a wide range of functions, including nutrient metabolism, detoxification, protein synthesis, and the production of biochemicals necessary for digestion [15]. To investigate the developmental methylation changes of m^6^A in liver, we generated transcriptome-wide m^6^A methylation maps at three postnatal developmental stages that have distinct diets: newborn piglets (0 day old) receiving nutrients through sow placenta, suckling piglets (21 days old) fed with breast milk of the mother sow and adult multiparous sows (2 years old) fed with balanced artificial diet. We characterized the developmentally transcriptome-wide m^6^A distribution patterns and analyzed the relationship between gene expression and m^6^A modification. We also identified extensively m^6^A-modified genes which may contribute to the differences of potential biological functions among three developmental stages with the distinct nutrient conditions. These results provide a resource for identifying adenosine methylation modified mRNAs in liver and extended our knowledge of the role of m^6^A in development and growth of mammalian organs.

## Materials & methods

### Animals and tissue collection

Three female pigs (Rongchang pig, a Chinese indigenous breed) for each of three postnatal developmental stages (i.e., newborn piglets, suckling piglets at 21 days old, and adults at 2 years old) raised on a Rongchang pig elite reservation farm in Chongqing were used in this study. They have different sources of diets: newborn piglets receiving nutrients through the sow placenta, suckling piglets fed with breast milk of the mother sow and two-year-old adults fed with balanced artificial diets. Animals were humanely killed to ameliorate suffering by intravenous injection with 2% pentobarbital sodium (25mg/Kg). Liver from each of the animal was separated rapidly from each carcass, and immediately frozen in liquid nitrogen and stored at -80°C until use. All experimental procedures and sample collection in this study were approved by the Institutional Animal Care and Use Committee (IACUC) of Sichuan Agricultural University, under permit No. DKY-B20141401.

### RNA preparation

High quality RNA from liver samples was isolated using Trizol according to the manufacturer’s instructions (Invitrogen). NanoDrop spectrophotometer (Thermo Scientific) was used to measure the concentration of RNA, and the integrity were tested by Agilent 2100 bioanalyzer. For isolation of poly(A) RNA, total RNA was subjected to two rounds of purification using oligo(dT)-coupled magnetic beads according to the manufacturer’s instructions (Ambion). Then, mRNA concentration was measured by Qubit 2.0 (Invitrogen) and the integrity was tested by agarose gel electrophoresis and Agilent 2100 bioanalyzer.

The mRNA was chemically fragmented approximately 150-nucleotide-long using NEBNext® Magnesium RNA Fragmentation Module (NEB #E6150S) according to the manufacturer's protocol. Standard ethanol precipitation was performed to precipitate the fragmented RNA. Size distribution of the RNA fragments was evaluated by agarose gel electrophoresis.

### RNA immunoprecipitation (RIP)

About 5 *μ*g fragmented mRNA was subjected to immunoprecipitation, according to the reported MeRIP method [16]. Briefly, fragmented RNA was incubated for 2 h at 4°C with 10 *μ*g m^6^A antibody (Synaptic Systems Cat. No.202003, diluted to 0.5 mg/ ml) in 1,000 *μ*l RIP buffer (50 mM Tris-HCl, 750 mM NaCl and 0.5% Igepal CA-630), supplemented with 2 mM RVC (Sigma) and 200 U RNasin (Promega). To reduce nonspecific binding, protein-A beads were pre-blocked in 1,000*μ*l RIP buffer with 0.5 mg/ml BSA for 2h at 4°C. The pre-blocked protein-A beads were then incubated with above mixture for another 2 h at 4°C. The beads were vigorously washed using 1,000 *μ*l RIP buffer three to four times. Discard the RIP buffer. Add 300 *μ*l dilution buffer (10 mM Tris-HCl pH 7.5) into the bead tube and incubate at 50°C for 90 min. Eluted RNA was precipitated by ethanol-NaAc solution and glycogen (Life Technologies) overnight at -80°C. The eluted RNA was treated with RNasin (Promega) according to the manufacturer’s instructions.

### Library preparation and high-throughput sequencing

NEBNext^®^ Ultra™ RNA Library Prep Kit for Illumina^®^ (E7530L, New England Biolabs) was used to construct the libraries from immunoprecipitated RNA and input RNA. In case that adaptor-ligated DNA population was evident in the library, size selection was performed using AMPure XP Beads according to the manufacturer’s instructions. Successful preparation of each library before proceeding to massively parallel deep sequencing was confirmed by Agilent 2100 Bioanalyzer and RT-PCR. Paired-end sequencing using standard 150 nucleotides read size was done with Illumina HiSeq 4000 sequencing platform. Raw sequencing data was processed by the Illumina base-calling pipeline.

### Data analysis

Adaptor and low-quality bases were trimmed with Skewer (version: 0.1.126) [17], and the clean reads were aligned to the reference pig genome (*Sscrofa10*.*2*) downloaded from Ensemble (www.ensembl.org) with BWA MEM (version:0.7.12) [16]. Duplicated reads were marked with Samblaster (version: 0.1.22) [18]. Only the uniquely mapped (MAPQ ≥ 13) and non-duplicated alignments were used for peak calling. MACS2 (version: 2.1.0.20150420) [19] was employed to perform peak calling with a threshold of *q* 0.05.

Peaks with ≥ 50% length overlap in at least two biological replicates were defined as high-confidence peaks and used for further analysis. The 101 nucleotides centered on the summits detected by MACS2 were used for detection of the consensus m^6^A motif by DREME (version: 4.10.2) [20]. Motif central enrichment was performed by CentriMo (version: 4.10.2) [21] with 301 nucleotides centered on the summits. To compare the positional distribution of the motif in the peaks, the top three RRm^6^ACH motifs and one false positive sequence are shown.

Fragments with no less than 50% partial overlap with peaks or with no less than 50% overlap with peak bases were counted with bedtools (version: 2.25.0) [22] and normalized to the total as fragments per million (FPM). The immunoprecipitation FPM was divided by the input FPM to calculate the signal enrichment of the peaks. Differential methylation was determined by Student’s t test (*P* < 0.05) in two stages. Higher methylation peaks of one stage was defined as the peaks with log_2_-transformed fold changes of peak enrichment > 0 or < 0, compared to the other two stages (Student’s t test, *P* < 0.05), plus peaks uniquely found in one stage. Gene expression was calculated by featureCounts (version:1.5.0-p3) [23] using input unique alignment reads. Differential expression was determined by DESeq2 (version: 1.12.4) with *P* < 0.05 [24]. Higher expression genes were defined as genes with FPKM (reads per kilo base of exon model per million mapped reads) log_2_-transformed fold changes > 0 or < 0, compared to other two stages (Student’s t test, *P* < 0.05). GO analysis was performed using DAVID (Database for Annotation, Visualization and Integrated 16 Discovery, version: 6.8) web server (https://david-d.ncifcrf.gov/) [25].

## Results

### MeRIP-seq summary

We performed a transcriptome-wide survey of m^6^A methylation in porcine liver at three developmental stages using the MeRIP-Seq technique [9, 16]. A total of 18 libraries consisting of three replicates of input and MeRIP samples from the three stages were sequenced (S1 Table). An average of 9.33 giga base-pair (Gb) of high-quality data for each MeRIP library and 7.67 Gb for each input library were generated. After removing reads aligned to multiple positions of the pig genome and duplicated reads derived from PCR artifacts, an average of 6.70 Gb for each MeRIP library and 5.86 Gb for each input library uniquely aligned to reference pig genome (*Sscrofa* 10.2). Reads of paired input and MeRIP libraries were used to identify peaks. For all three replicates, 32,661 distinct narrow m^6^A peaks from newborn, 25,921 from suckling and 28,848 from adult stages were successfully detected in liver transcriptomes, which harbor an average of 13,578 transcribed genes (S1 Table).

### General features of m^6^A methylation

**Fig 1:**
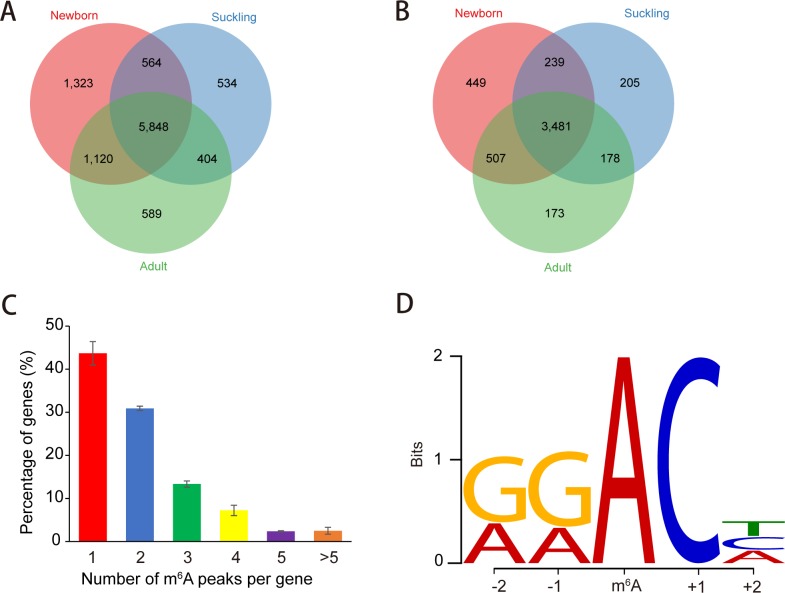
Overview of m^6^A methylation in porcine liver. (A) Venn diagram showing the overlap of m^6^A peaks in newborn (8,855), suckling (7,350) and adult (7,961). There are 5,848 common peaks among the three stages, which with ≥ 50% length overlap between stages. (B) Venn diagram showing the overlap of m^6^A modified genes. Respectively, 4,676 genes in newborn, 4,103 in suckling and 4,339 in adult were m^6^A methylated. For all three stages, 3,481 genes were consistently modified. (C) Proportion of genes containing variant numbers of m^6^A peaks. Majority of modified genes (74.60%) contain one or two m^6^A peaks, while the rest contains more. (D) Sequence logo representing the most common consensus motif (RRm^6^ACH) in the m^6^A peaks. The consensus sequence was detected by DREME (version: 4.10.2), using the 101 nucleotides centered on the summits of called original narrow peaks.

After merging the three replicates, we identified a total of 11,022, 8,727 and 9,860 distinct peaks in newborn, suckling and adult stages, respectively. On average, over 80% of the identified peaks were consistently detected in at least two biological replicates of each stage (S1A Fig) which confirmed the high reproducibility of MeRIP-seq among biological replicates (S1B Fig). We detected 8,855 high-confidence m^6^A peaks in newborn, 7,350 in suckling, and 7,961 in adult stages (S2 Table). We used the recurrent peaks as high-confidence m^6^A peaks for subsequent analysis (S3 Table). Consequently, 5,848 peaks were consistently detected within all three stages (Fig 1A). On average, 74.24% of the identified high-confidence peaks overlapped with intragenic regions, representing transcripts of 4,676, 4,103 and 4,339 genes in the newborn, suckling and adult developmental stages, respectively (S2 Table). For all three stages, 3,481 genes were consistently modified by m^6^A (Fig 1B and S4 Table). Our results showed that about one-third of expressed genes were modified by m^6^A, with 35.09%, 30.40%, and 32.61% of expressed genes (FPKM > 0.1) detected in the newborn, suckling and adult stages, respectively (S2 Table). The results also showed that the liver transcriptome contains about 1.33 to 1.42 m^6^A peaks per m^6^A modified genes (S2 Table).

The number of m^6^A modified sites varied from 1 to 14 among individual genes, while 74.60% of modified genes harbor only one or two m^6^A peaks. The remaining genes (25.40%) contain three or more peaks (Fig 1C), which is much higher than that previously reported for human brain (16.70%) [10].

The classic consensus sequence, RRm^6^ACH, where R represents a purine and H represents a non-guanine base [26, 27] was found in most (78.90%) of the detected narrow peaks (S5 Table). The consensus sequence (Fig 1D) observed in the current study indicated conserved m^6^A methylation among different species. The most frequent two motifs were GGm^6^ACC (21.99%) and GGm^6^ACT (21.80%) (Fig 1D). We further performed a motif central enrichment analysis using CentriMo (version: 4.10.2) [21], and found motifs detected in peaks were mainly enriched around the summits of called narrow peaks and at the center of merged peaks (S2 Fig).

### Topological pattern of m^6^A methylation

**Fig 2:**
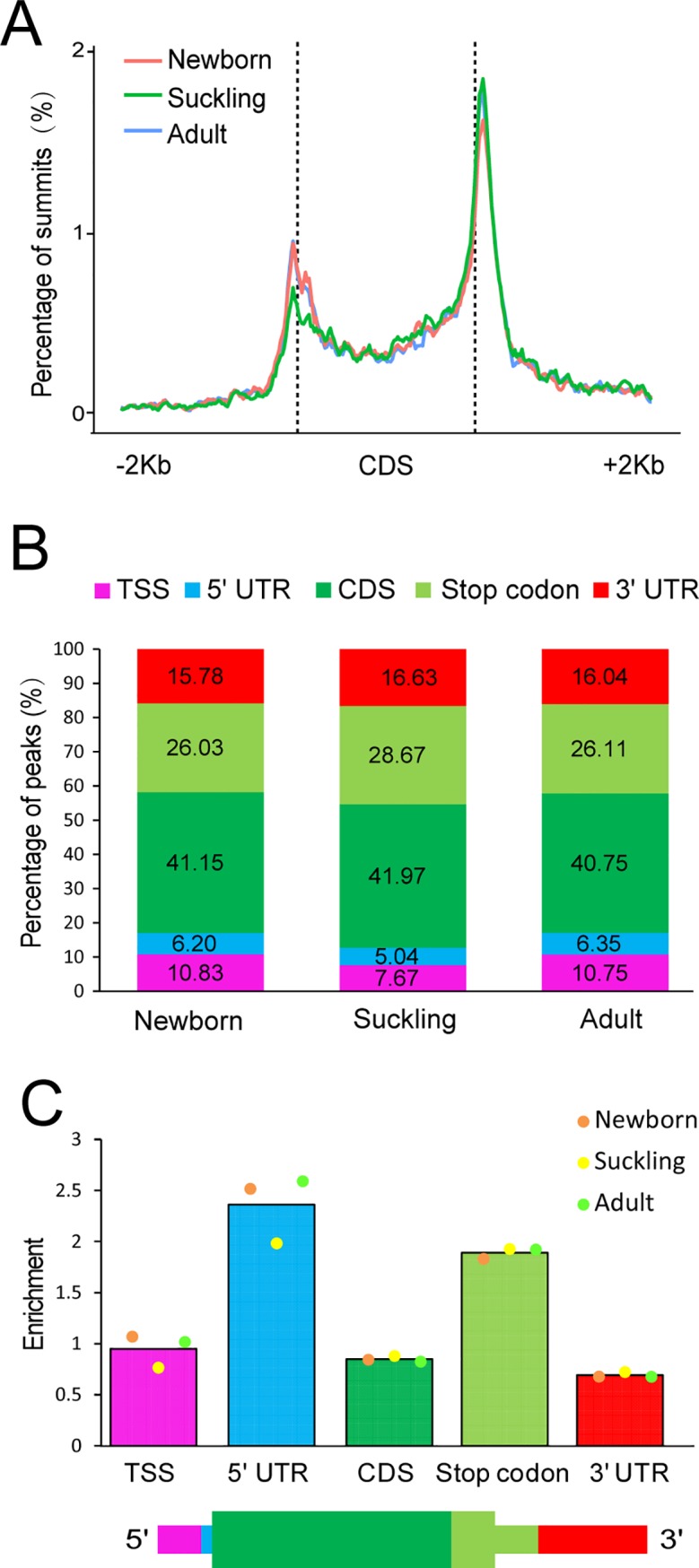
Distribution pattern of m^6^A peaks. (A) Distribution of summits of m^6^A peaks along transcripts. Each transcript was divided into three parts: -2Kb, CDS, +2Kb. Each part was divided into 100 bins, and the percentage of m^6^A summits of each bin was determined. Moving averages (4 bins) of summit percentage of newborn (red), suckling (green) and adult (blue) are shown. (B) Graphical representation of frequency of m^6^A peaks in five non-overlapping segments of three stages (TSS: 200 nucleotides downstream of the TSS, stop codon: a 400 nucleotide window centered on the stop codon). m^6^A peaks were most abundant in CDS and stop codon segments. (C) Top, relative enrichment of m^6^A peaks across transcript segments, normalized by the relative fraction that each segment occupies in the transcriptome. Bottom, schematic of the five segments. 5' UTR and stop codon were the most enriched segments after normalization.

To understand the topological pattern of m^6^A methylation in liver transcriptome, we investigated the distribution profiles of m^6^A peaks. Consistent with previous studies in mouse and human [6, 9], we observed a significant enrichment of m^6^A peaks around the start and stop codons of transcripts in all three stages (Fig 2A). To further confirm the preferential localization of m^6^A along transcripts, we categorized m^6^A peaks into five non-overlapping segments: TSS (200 nucleotides downstream of the TSS), 5' untranslated region (UTR), coding sequence (CDS), stop codon segment (a 400 nucleotide window centered on the stop codon) and 3' UTR. m^6^A peaks were most abundant in CDS (40.75% to 41.97%) and stop codon segments (26.03% to 28.67%), followed by the 3' UTR, TSS and then 5' UTR segments (Fig 2B). After segment normalization according to relative fraction of the transcriptome occupied by each segment, we observed that the 5' UTR and stop codon were the most enriched segments (Fig 2C).

### Relationship between m^6^A methylation and gene expression

**Fig 3:**
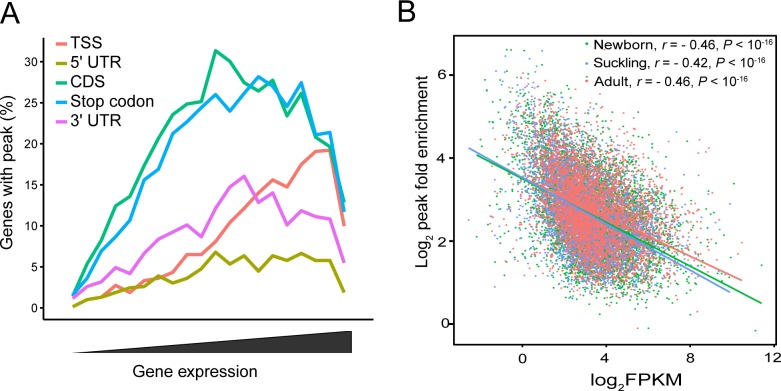
Relationship between m^6^A methylation and expression of modified genes. (A) Fraction of genes with m^6^A peaks in each of the segments as a function of expression level. Most of the modified genes were expressed at moderate levels. Genes expressed at the two extremes were less methylated. (B) Plot of m^6^A peak enrichment and mRNA abundance in the three stages. Obvious negative correlation between m^6^A peak enrichment and modified mRNA abundance was found (Pearson’s *r* = -0.47 to -0.42, *P* < 10^−16^). Lines represent the linear trend for the obtained values.

We next determined whether gene expression in porcine liver is correlated with the presence of m^6^A modification by plotting the fraction of genes with m^6^A peaks in each of the segments as a function of expression level (Fig 3A). Each segment showed a similar non-monotonic pattern as previously exhibited in a mouse and human study [9]. The non-monotonic pattern showed that most m^6^A modified genes were expressed at moderate levels (Fig 3A).

A plot of m^6^A peak enrichment level versus mRNA abundance revealed a negative correlation between enrichment and gene expression in all three stages (Pearson’s *r* = -0.47 to -0.42, *P* < 10^−16^) (Fig 3B), which is consistent with previous reports [10, 14]. A more detailed, distribution pattern-based analysis also showed negative correlations between m^6^A peak enrichment and gene expression for all five segments (S3 Fig). Among them, stop codon (average Pearson’s *r =* -0.50, *P* < 10^−16^) and CDS (average Pearson’s *r* = -0.47, *P* < 10^−16^) segments showed the highest negative correlation, and relatively lower correlation rates were found for 3' UTR (average Pearson’s *r* = -0.42, *P* < 10^−16^), 5' UTR (average Pearson’s *r* = -0.33, *P* < 10^−7^) and TSS (average Pearson’s *r* = -0.28, *P* < 10^−6^) segments (S3 Fig).

### m^6^A modified genes involve in biologically important pathways

**Fig 4:**
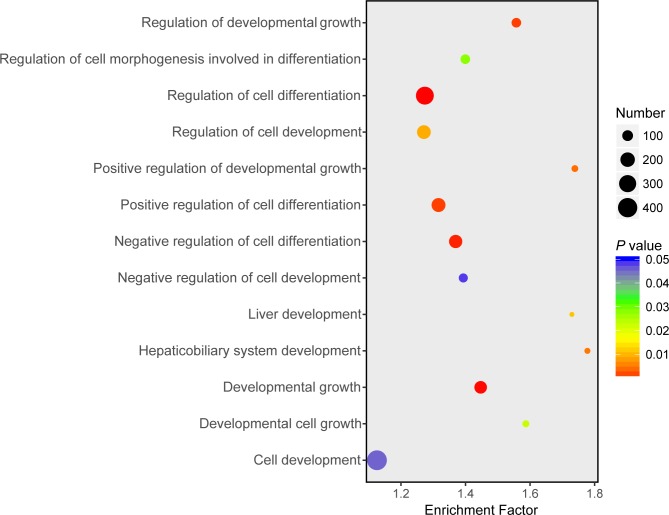
Cell differentiation and liver development related GO categories enriched for genes modified by m^6^A methylation. Some genes consistently modified by m^6^A methylation in all three stages were specifically involved in cell differentiation- and liver development- related categories. Different colors represent *P* values, and sizes represent gene numbers. *X*-axis represents fold enrichment. Detailed functional enrichment analysis results of all consistently modified genes, are available in S6 Table.

To assess the function of m^6^A in porcine liver, 3,481 genes that were consistently modified by m^6^A methylation in all three stages were subjected to gene functional enrichment analysis using DAVID tool (version: 6.8). As a result, these genes were significantly (*P* < 0.05, Benjamini-Hochberg corrected) enriched in a variety of cellular functions relevant to “RNA metabolic process” (951 genes), “regulation of transcription” (745 genes), “regulation of signal transduction” (581 genes) and “biosynthetic process” (1045 genes) (S6 Table). In addition, some genes were specifically involved in cell differentiation- and liver development- related categories, such as “regulation of cell differentiation” (333 genes), “hepaticobiliary system development” (42 genes) and “liver development” (40 genes) (Fig 4).

### Differential m^6^A modification and gene expression

An average of 34.94% of m^6^A modified genes showed differential methylation, and an average of 17.19% of expressed genes were differentially expressed between two stages (Table 1). A paired analysis of differential methylation and differential expression showed that liver of newborn pigs had the largest m^6^A differential methylation ratio (average 25.58%) and the smallest differential expression ratio (average 4.76%) among the three stages, while liver of the suckling pigs showed an opposite extreme (Table 1). This result further confirmed the negative correlation between m^6^A methylation and gene expression.

**Table 1:** Number of genes showing differential transcript levels and differential m^6^A methylation.

		Newborn vs. suckling		Newborn vs. adult		Suckling vs. adult	
		Higher in newborn	Higher in suckling	Higher in newborn	Higher in adult	Higher in suckling	Higher in adult
**Differential m**^**6**^**A methylation**	Genes (n)	1,513	488	1,068	530	607	994
	Proportion (%)	29.91	9.65	21.25	10.54	12.69	20.78
	Total (%)	39.56		31.79		33.47	
**Differential gene expression**	Genes (n)	713	1654	725	1,413	1743	1,498
	Proportion (%)	4.70	10.90	4.81	9.37	11.71	10.07
	Total (%)	15.6		14.18		21.78	

Differential m^6^A methylation and differential gene expression were determined by Student’s t test (*P* < 0.05) between stages. Higher methylation: peak log_2_-transformed fold changes > 0 or <0, *P* <0.05, plus peaks uniquely found in this stage. Higher expression: FPKM log_2_-transformed fold changes > 0 or < 0, *P* <0.05.

**Fig 5:**
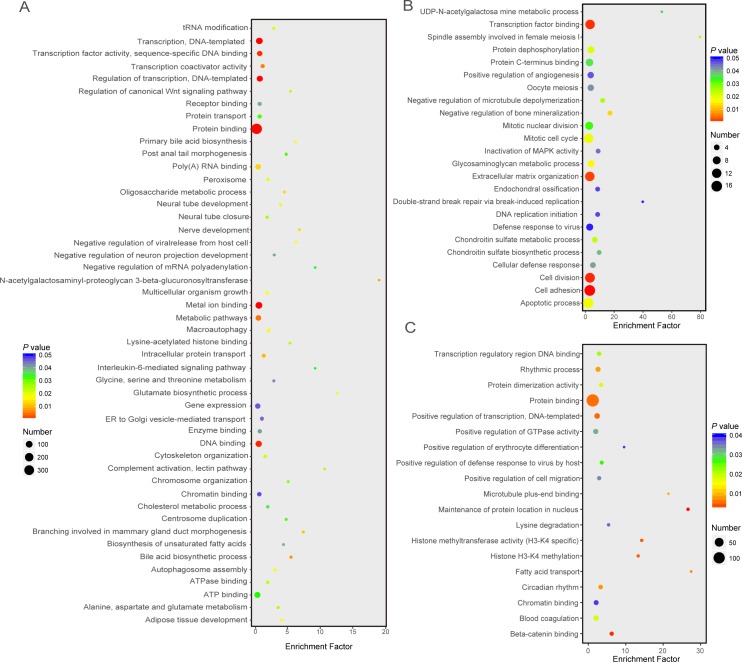
**GO terms of genes showing a higher enrichment of m**^**6**^**A methylation in newborn (A), suckling (B) and adult (C).** Different colors represent *P* values, and sizes represent gene numbers. *X*-axis represents fold enrichment.

Analysis of differentially m^6^A modified genes showed that 748 genes (16.00%) in newborn, 275 (6.70%) in suckling, and 280 (6.45%) in adult stages were more highly enriched for m^6^A peaks compared with the other two stages (S4 Fig). These genes exhibited much higher negative correlations between gene expression and m^6^A peak enrichment (average Pearson’s *r* = -0.56, *P* < 10^−16^) compared with correlations between overall peak enrichment and gene expression (S5 Fig). Based on gene functional enrichment analysis, genes showing higher m^6^A methylation at the newborn stage were significantly (*P* < 0.05) enriched to bile acid secretion and nutrients metabolic processes, such as “bile acid biosynthetic process” (5 genes), “oligosaccharide metabolic process” (5 genes) and “cholesterol metabolic process” (7 genes) GO categories, “alanine, aspartate and glutamate metabolism” (5 genes), “biosynthesis of unsaturated fatty acids” (4 genes) and “glycine, serine and threonine metabolism” (5 genes) pathways (Fig 5A). Genes with higher m^6^A methylation at the suckling stage were related to “glycosaminoglycan metabolic process” (6 genes), “UDP-N-acetylgalactosamine metabolic process” (2 genes) GO categories and the “oocyte meiosis” (5 genes) pathway (Fig 5B). Genes with higher m^6^A methylation at the adult stage were related to “fatty acid transport” (3 genes), “circadian rhythm” (9 genes) and “lysine degradation” (4 genes) categories or pathways (Fig 5C).

**Fig 6:**
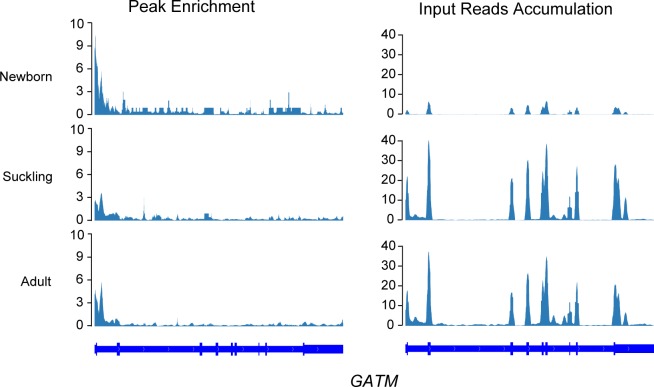
m^6^A enrichment and gene expression profile of *GATM* in three stages. Opposite trends of the m^6^A methylation level (left panel) and gene expression level (right panel) of *GATM* are shown. Gene expression level is presented by the accumulation of input reads.

Having shown a negative correlation between m^6^A methylation and gene expression, we next explored functions of genes with relatively high m^6^A methylation levels and low levels of expression. We found that 31 genes in the newborn stage and three in the adult stage simultaneously showed higher m^6^A methylation and lower expression compared with the other two stages (S7 Table). The genes in the newborn stage were potentially expressed for organic acid biosynthetic and metabolic processes, such as “oxoacid metabolic process”, “folic acid-containing compound metabolic process” and “glycine, serine and threonine metabolism” (Table 2). Typically, *GATM* shows the highest methylation at the newborn stage and the lowest at the adult stage (Fig 6), which mainly involves in amino acid and organic acid metabolism. In contrast, the lowest expression of *GATM* was at the newborn stage and highest at the adult stage. Only three genes (*EDEM2*, *MAFK*, *UBALD2*) were detected with higher m^6^A methylation and lower expression at the adult stage. These genes involved in “mannosyl-oligosaccharide 1,2-alpha-mannosidase activity” (*EDEM2*) and “transcription factor activity” (*MAFK*) (Table 2).

**Table 2:** Functions of genes with higher m^6^A peak enrichment and lower expression.

Stages	Functions^a^	Gene symbol	Reference^b^
**Newborn**	Cellular amino acid metabolic process	*HOGA1*, *DPYS*, *FOLR1*, *GATM*, *ALDH4A1*, *SARDH*, *GSTZ1*, *BAAT*, *GNMT*	[28–30]
	Carboxylic and oxoacid acid metabolic process	*HOGA1*, *DPYS*, *FOLR1*, *GATM*, *CYP1A2*, *ALDH4A1*, *CYB5R3*, *SARDH*, *GSTZ1*, *BAAT*, *GNMT*	[28, 31]
	Cofactor metabolic process	*FOLR1*, *CYP1A2*, *SARDH*, *BAAT*, *GNMT*	[28, 31]
	4-hydroxyproline catabolic process	*HOGA1*, *ALDH4A1*	[30, 32]
	Steroid metabolic process	*CYP1A2*, *CYB5R3*, *BAAT*, *APOF*	[28]
	Single-organism catabolic process	*HOGA1*, *DPYS*, *CYP1A2*, *ALDH4A1*, *XDH*, *GSTZ1*, *VAMP8*	[28, 31]
	Coenzyme metabolic process	*FOLR1*, *SARDH*, *BAAT*, *GNMT*	[28]
	Carboxylic acid biosynthetic process	*HOGA1*, *GATM*, *ALDH4A1*, *BAAT*	[28]
	Dicarboxylic acid metabolic process	*HOGA1*, *FOLR1*, *ALDH4A1*	[28]
	Oxidation-reduction process	*CYP1A2*, *ALDH4A1*, *CYB5R3*, *SARDH*, *XDH*, *GNMT*, *MARC2*	[28, 29, 31, 33]
	Folic acid-containing compound metabolic process	*FOLR1*, *SARDH*	[34]
	Metabolic pathways	*HOGA1*, *DPYS*, *GATM*, *CYP1A2*, *ALDH4A1*, *SARDH*, *XDH*, *GSTZ1*, *BAAT*, *PCK2*	[28, 31]
	Glycine, serine and threonine metabolism	*GATM*, *SARDH*, *GNMT*	[28]
	Arginine and proline metabolism	*HOGA1*, *GATM*, *ALDH4A1*	[28]
	Caffeine metabolism	*CYP1A2*, *XDH*	[31]
	Ovarian and testicular apolipoprotein	*APON*	
	Complement and coagulation cascades	*C1R*, *C1QA*	[35]
	Lysosome	*LAPTM4B*	
	Polyunsaturated fatty acids binding	*AZGP1*	[36]
	Insulin-like growth factor binding	*IGFALS*	[37]
	Receptor binding and beta-catenin binding	*SLC9A3R2*	[38]
	Other binding	*ARVCF*, *C7orf50*	
	ATP-dependent peptidase activity	*LONRF3*	
	Bile secretion	*KCNN2*	[39]
	Nucleic acid binding and ribonuclease activity	*RNASE4*	[40]
	Enzyme protein C-terminus binding	*ECM1*	[41]
	Selenide, water dikinase activity	*SEPHS2*	
**Adult**	Mannosyl-oligosaccharide 1,2-alpha-mannosidase activity	*EDEM2*	[42]
	Transcription regulatory region sequence-specific DNA binding	*MAFK*	[43]
	Unkonwn	*UBALD2*	

^a^Suggests the function of proteins expressed by m^6^A modified genes.

^b^The functions of many genes were inferred by gene ontology (GO) analysis using DAVID and some functions were inferred from publications.

## Discussion

This study reports comprehensive transcriptome-wide patterns of m^6^A in porcine liver, based on a previously reported MeRIP-seq method [9, 16]. We report abundant m^6^A sites in the porcine liver transcriptome with a density of 1.33–1.42 site per gene, which is comparable with that obtained in mouse liver (~1.34 m^6^A sites per coding gene). We profiled features and patterns of m^6^A, including the extent of m^6^A gene modification, m^6^A distribution in transcripts, and occurrence of the consensus m^6^A methylation motif. All showed high concordance with m^6^A characteristics of previous reports [9, 10], indicating conserved RNA adenosine methylation between pig and other species.

Most of the m^6^A modified genes were expressed at a medium level, and a negative correlation was found between m^6^A peak enrichment and gene expression (Pearson’s *r* = -0.47 to -0.42, *P* < 10^−16^). m^6^A is a chemical mark associated with transcript turnover. m^6^A-marked transcripts have a significantly shorter RNA half-life and increased rates of mRNA decay compared to non-m^6^A-marked transcripts [14]. Meanwhile, a high level of m^6^A methylation may endow transcripts expressed at low levels with high RNA stability, or provide a signal for reader protein binding [9, 44]. In addition, we noted that over 25% of m^6^A-marked genes harbor three or more m^6^A sites, which may increase RNA stability or probability targeted by m^6^A readers. These results indicate that m^6^A methylation mediates a level of post-transcriptional regulation of gene expression.

We found that genes involved in hepatic cell differentiation and liver development were consistently modified by m^6^A methylation in the three postnatal developmental stages. Previous studies have analogous results. For instance, m^6^A methylation in embryonic stem cells regulates core pluripotency factors involved in development and differentiation [14]. Depletion of m^6^A levels results in embryonic stem cells advancing from self-renewal toward differentiation to specific lineages [14, 45, 46]. In *Arabidopsis*, a reduction of m^6^A can impair early embryonic development at the globular stage [47]. Recent studies of the differential methylation of m^6^A in three different organs of *Arabidopsis* revealed that m^6^A is an important contributor to organ differentiation [7]. Therefore, m^6^A may be an important conserved regulator of cell differentiation and development in both animals and plants.

The liver plays an important role in metabolism, with numerous functions including the regulation of glycogen storage, plasma protein synthesis, decomposition of red blood cells, hormone production, and detoxification [15]. In this study, we found that at any one stage, genes with higher levels of m^6^A methylation had metabolic functions required for or specific to this stage. The newborn liver mainly metabolizes nutrients obtained through the placenta from the sow and synthesizes macromolecules that are necessary for rapid growth [41–43]. Functional enrichment analysis of genes with higher m^6^A methylation at this stage produced terms such as “metabolic pathways”, “biosynthesis of unsaturated fatty acids”, “bile acid biosynthesis process” and “oligosaccharide metabolic process”. In the suckling stage, the liver mainly metabolizes nutrients from breast milk, which is enriched in carbohydrates and protein, including total solids, fat, lactose, total protein, total whey protein and individual immunoglobulin classes [48]. Interestingly, genes in this stage with higher m^6^A methylation were enriched for the terms “UDP-N-acetylgalactosamine metabolic process”, “glycosaminoglycan metabolic process” and other monosaccharide or glycoprotein metabolic processes. In the adult stage, the liver has a fully developed hepaticobiliary system compared with the other two stages [49]. Nutrients at this stage are mainly derived from a nutritionally balanced artificial diet [50], which contains mainly starch, protein, fat, and some additives, such as lysine and threonine [51, 52]. Genes with higher m^6^A methylation at this stage were mainly related to metabolic activities, such as “lysine degradation”, “regulation of catalytic activity”, “fatty acid transport” and “positive regulation of metabolic process”, which correspond to adult metabolic functions of the liver. In addition, terms such as “rhythmic process” and “circadian rhythm” were also enriched for highly m^6^A-modified genes in the adult stage, indicating circadian clock control of liver metabolic functions [53]. Therefore, highly m^6^A-methylated genes at a particular stage showed enriched terms that were consistent with liver function at this stage.

Genes with higher levels of m^6^A methylation and lower levels of expression also showed close association with metabolic activities in each stage. In newborn liver, genes were mainly involved in metabolic processes of organic acids, such as carboxylic acid, oxoacid and dicarboxylic acid, indicating that m^6^A methylation plays roles in balancing post-transcription expression levels of genes involved in central metabolic processes. For example, *GATM* and *GNMT* had higher levels of m^6^A methylation and lower levels of expression in newborn and are involved in amino acid metabolism [29] and S-adenosylmethionine (SAM) synthesis [54]. *GATM* encodes the first and rate-limiting product in creatine biosynthesis, and creatine and its phosphorylated form play essential roles in energy metabolism [55]. *GNMT* is a potential tumor suppressor that is commonly inactivated in human hepatoma. *GNMT* affects transmethylation kinetics and SAM synthesis by limiting homocysteine remethylation fluxes [54]. Cytochrome P450 family 1 subfamily A member 2 (*CYP1A2*) encodes a member of the cytochrome P450 superfamily of enzymes involved in the metabolism of a variety of compounds, including steroids, fatty acids, and xenobiotics [31]. m^6^A methylation of *CYP1A2* was less and expression higher in suckling and adult stages, compared with the newborn. Expression of *CYP1A2* appears to be induced by dietary constituents [56]. In suckling and adult stages, expression of *CYP1A2* may be induced by various dietary constituents [56], especially the artificial diet in the adult stage, which contains a high content of polycyclic aromatic hydrocarbons (PAHs) [57, 58]. CYP1A2 mainly catalyzes conversion of PAHs to more polar and water-soluble metabolites, and the resultant metabolites are readily excreted from the body [59]. In the newborn stage, *CYP1A2* expression is relatively low, as it is not induced by such dietary constituents. Higher m^6^A methylation may endow the relatively fewer transcripts with enough stability to perform their function. In the adult stage, the highly m^6^A modified and lowly expressed gene, *EDEM2*, mainly initiates ER-associated glycoprotein degradation by catalyzing the first mannose trimming step [60], which may play important roles in metabolism of glycoprotein of deriving from artificial diet.

In conclusion, we characterized diverse patterns of m^6^A in genes expressed in the porcine liver and showed that these genes act as important regulators in three developmental stages. The high negative correlation between levels of m^6^A methylation and modified gene expression suggests that adenosine methylation plays important biological roles in negative regulation of post-transcriptional gene expression. The m^6^A modified genes were mainly involved in regulation of differentiation and development of the porcine liver. As growing conditions and diets change in the three developmental stages, the liver undergoes different stimuli and nutrient levels, which may influence the differential expression of the transcriptome and differential m^6^A methylation of the epitranscriptome.

## Supporting information

S1 Fig High reproducibility of MeRIP-seq. (A) Distribution of peaks in three biological replicates across three stages. On average, 80% of peaks were shared by at least two replicates. (B) Heat map of Pearson’s correlation of read count of transcripts in both immunoprecipitation (MeRIP) and input data across nine samples.
(TIF)

S2 Fig **Central enrichment of consensus RRm**^**6**^**ACH motif sequences around m**^**6**^**A peak summits (A) and peak center of merged m**^**6**^**A peaks (B).** Top three consensus RRm^6^ACH motif sequences (GGACT/A/C) and one false positive sequence (GCAGC) were discovered by DREME, using the 101 nucleotides centered on the summits of called original narrow peaks. Motif central enrichment was performed by CentriMo (version: 4.10.2) with 301 nucleotides centered on the summits or peak center of merged m^6^A peaks. Each curve shows the density (averaged over bins of 40 bp width) of the best strong site (score ≥ 5 bits) for the named motif at each position in the m^6^A peak regions (301 bp). The legend shows the motif, its central enrichment *P*-value, the width of the most enriched central region (w), and the number of peaks (n out of 70,131 summits in (A), n out of 8,379 merged peaks in (B)) that contain a motif site. This similar tendency of central enrichment of RRm^6^ACH motifs suggested that we used a reliable merging process to deal with peaks in multiple biological replicates and groups.
(TIF)

S3 Fig Plot of m^6^A peak enrichment and mRNA abundance in five non-overlapping segments. Higher negative correlation rates were found in stop codon (average Pearson’s *r* = -0.50, *P* < 10^−16^) and CDS (average Pearson’s *r* = -0.47, *P* < 10^−16^) peaks compared with UTR and TSS peaks. Lines represent the linear trend for the obtained values.
(TIF)

S4 Fig **Venn diagram of paired comparison of genes with higher methylation among stages (A), paired comparison of genes with lower expression among stages (B), and overlap of genes with higher methylation and lower expression in each stage (C).** “N” represents newborn, “S” represents suckling and “A” represents adult. “>” represents higher methylation between stages and “<” indicates lower gene expression.
(TIF)

S5 Fig Plot of m^6^A enrichment and mRNA abundance of genes with higher m^6^A methylation compared with each of the other two stages. A higher negative correlation rate was found in all three stages (average Pearson’s *r* = -0.56, *P* < 10^−16^).
(TIF)

S1 Table Summary of sequenced and mapped data of the MeRIP-Seq and input RNA-seq samples. (DOCX)

S2 Table Number of expressed genes, merged peaks and proportion of m^6^A modified transcripts in each group. (DOCX)

S3 Table Lists of m^6^A peaks in three stages. (XLSX)

S4 Table Persistently m^6^A modified genes in the three developmental stages. (XLSX)

S5 Table Diverse patterns of m^6^A motif sequences (RRm^6^ACH). (DOCX)

S6 Table Functional categories of consistently modified genes in porcine liver of the three developmental stages. (XLSX)

S7 Table Genes showing higher m^6^A peak enrichment and lower expression levels than the other two stages. (XLSX)
