# Supplementary figures and images for: mRNA N6-methyladenosine methylation of postnatal liver development in pig
Source: PLoS One. 2017 Mar 7;12(3):e0173421. doi: 10.1371/journal.pone.0173421 (PMC5340393; doi:10.1371/journal.pone.0173421)

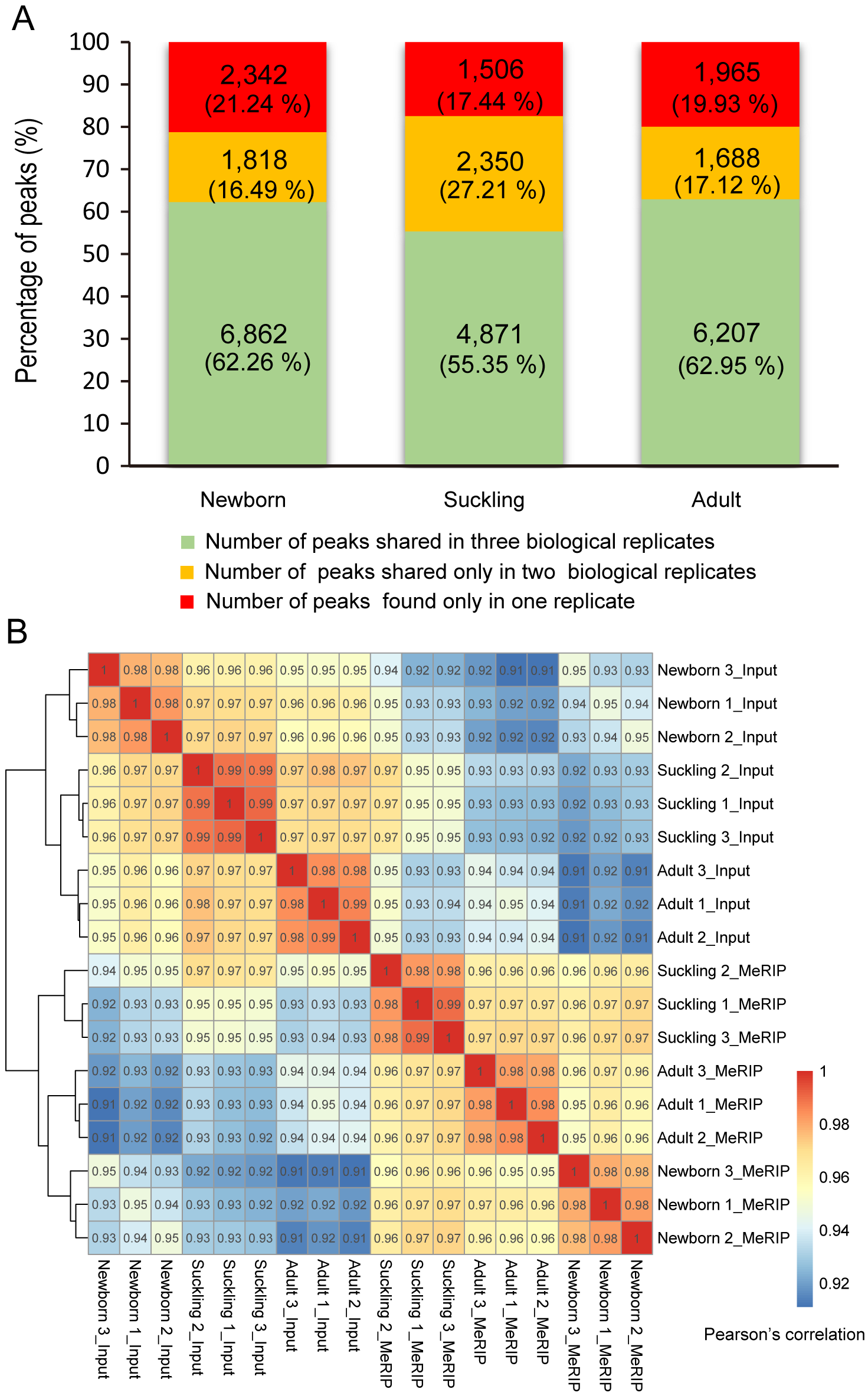

Supplement: S1 Fig — (A) Distribution of peaks in three biological replicates across three stages. On average, 80% of peaks were shared by at least two replicates. (B) Heat map of Pearson’s correlation of read count of transcripts in both immunoprecipitation (MeRIP) and input data across nine samples. (TIF) [file pone.0173421.s001.tif]

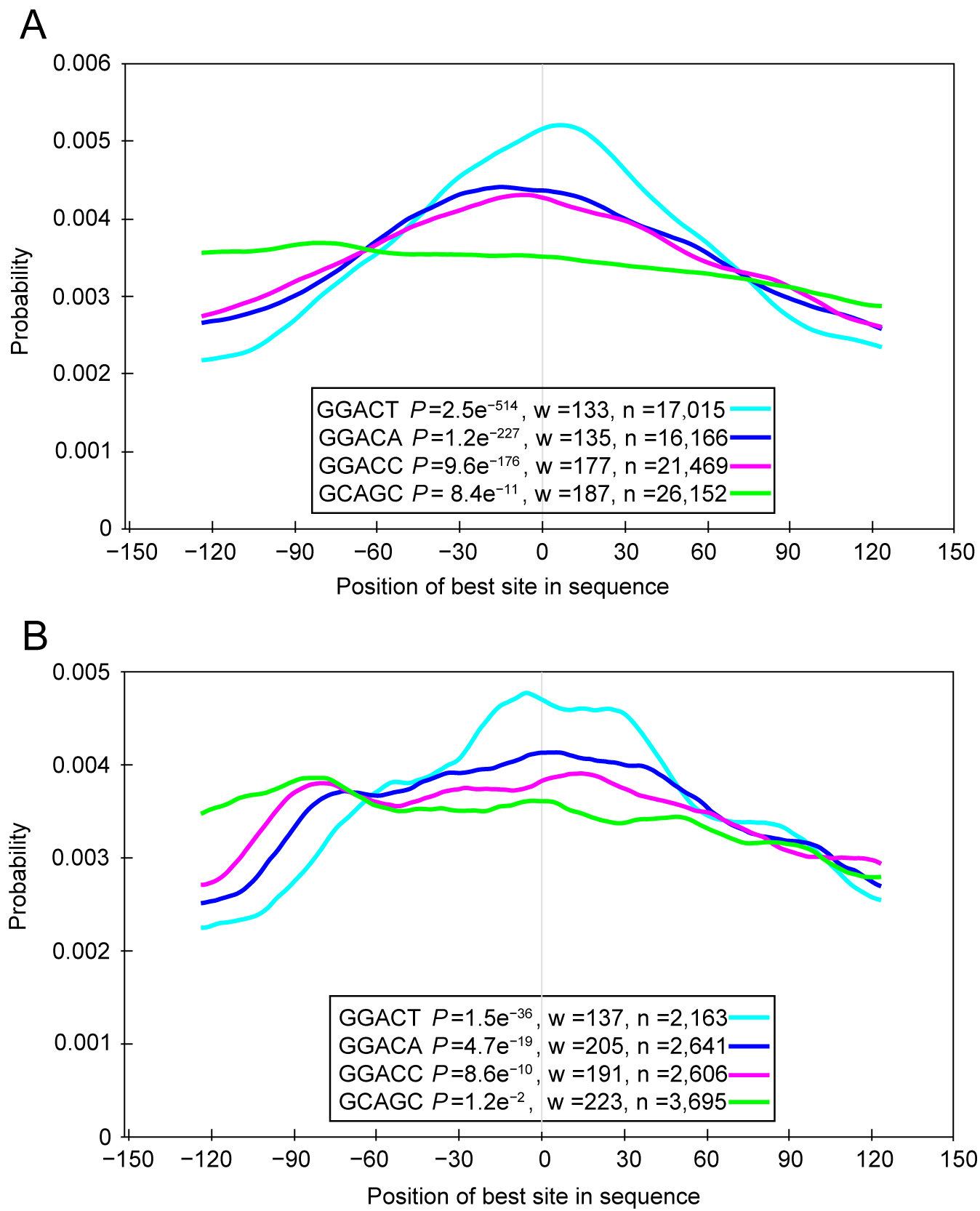

Supplement: S2 Fig — Central enrichment of consensus RRm6ACH motif sequences around m6A peak summits (A) and peak center of merged m6A peaks (B). Top three consensus RRm6ACH motif sequences (GGACT/A/C) and one false positive sequence (GCAGC) were discovered by DREME, using the 101 nucleotides centered on the summits of called original narrow peaks. Motif central enrichment was performed by CentriMo (version: 4.10.2) with 301 nucleotides centered on the summits or peak center of merged m6A peaks. Each curve shows the density (averaged over bins of 40 bp width) of the best strong site (score ≥ 5 bits) for the named motif at each position in the m6A peak regions (301 bp). The legend shows the motif, its central enrichment P-value, the width of the most enriched central region (w), and the number of peaks (n out of 70,131 summits in (A), n out of 8,379 merged peaks in (B)) that contain a motif site. This similar tendency of central enrichment of RRm6ACH motifs suggested that we used a reliable merging process to deal with peaks in multiple biological replicates and groups. (TIF) [file pone.0173421.s002.tif]

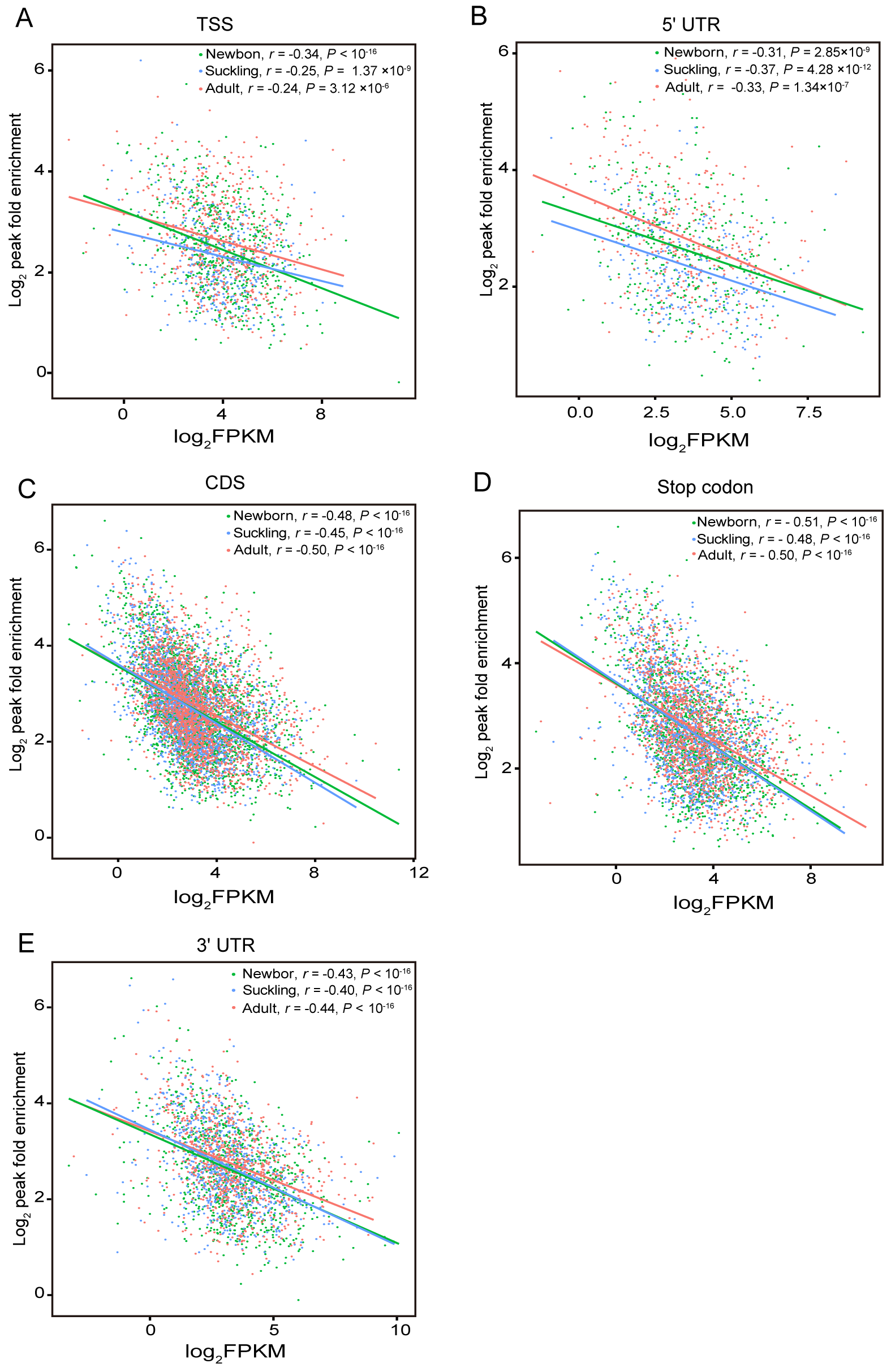

Supplement: S3 Fig — Higher negative correlation rates were found in stop codon (average Pearson’s r = -0.50, P < 10−16) and CDS (average Pearson’s r = -0.47, P < 10−16) peaks compared with UTR and TSS peaks. Lines represent the linear trend for the obtained values. (TIF) [file pone.0173421.s003.tif]

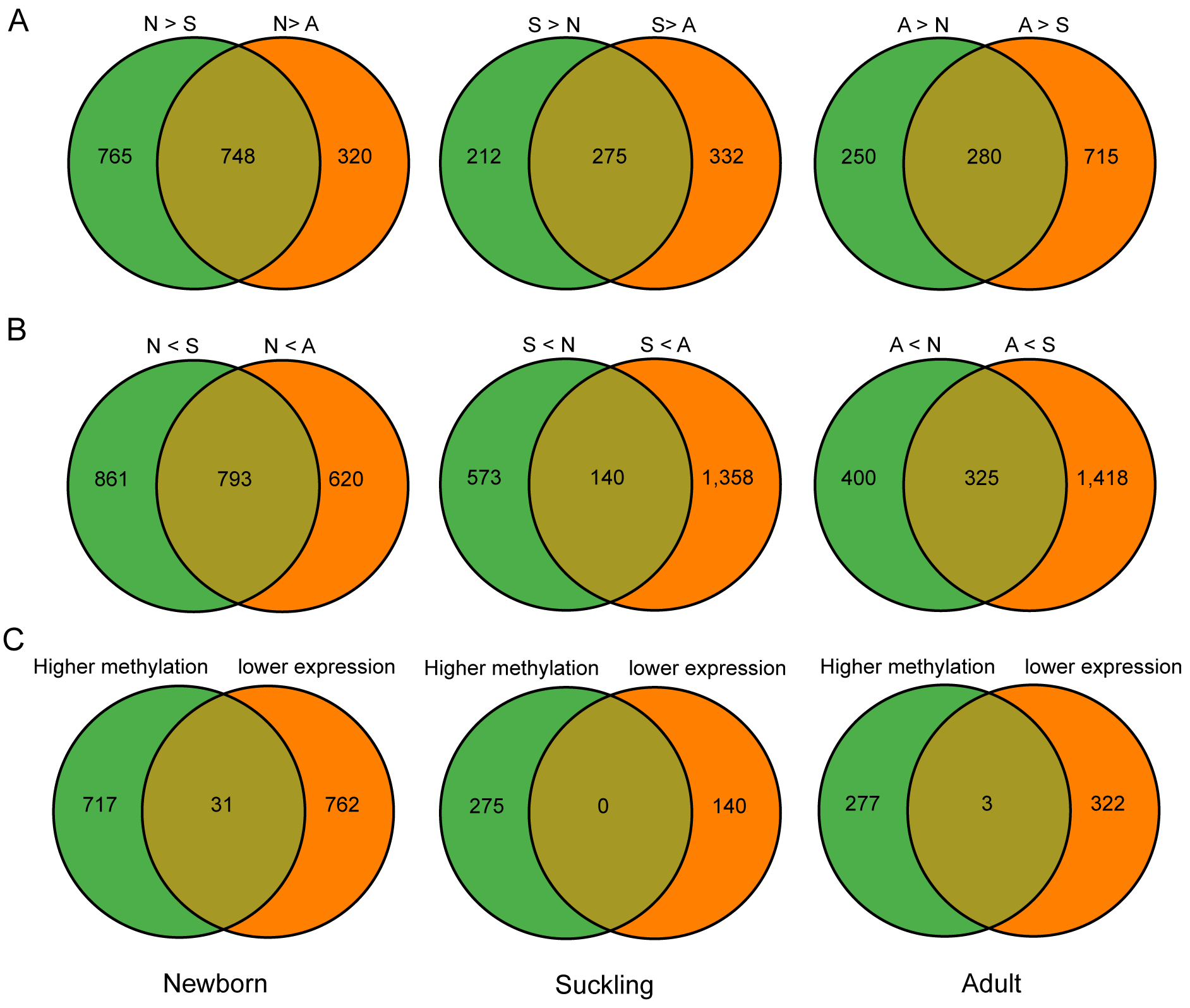

Supplement: S4 Fig — Venn diagram of paired comparison of genes with higher methylation among stages (A), paired comparison of genes with lower expression among stages (B), and overlap of genes with higher methylation and lower expression in each stage (C). “N” represents newborn, “S” represents suckling and “A” represents adult. “>” represents higher methylation between stages and “<” indicates lower gene expression. (TIF) [file pone.0173421.s004.tif]

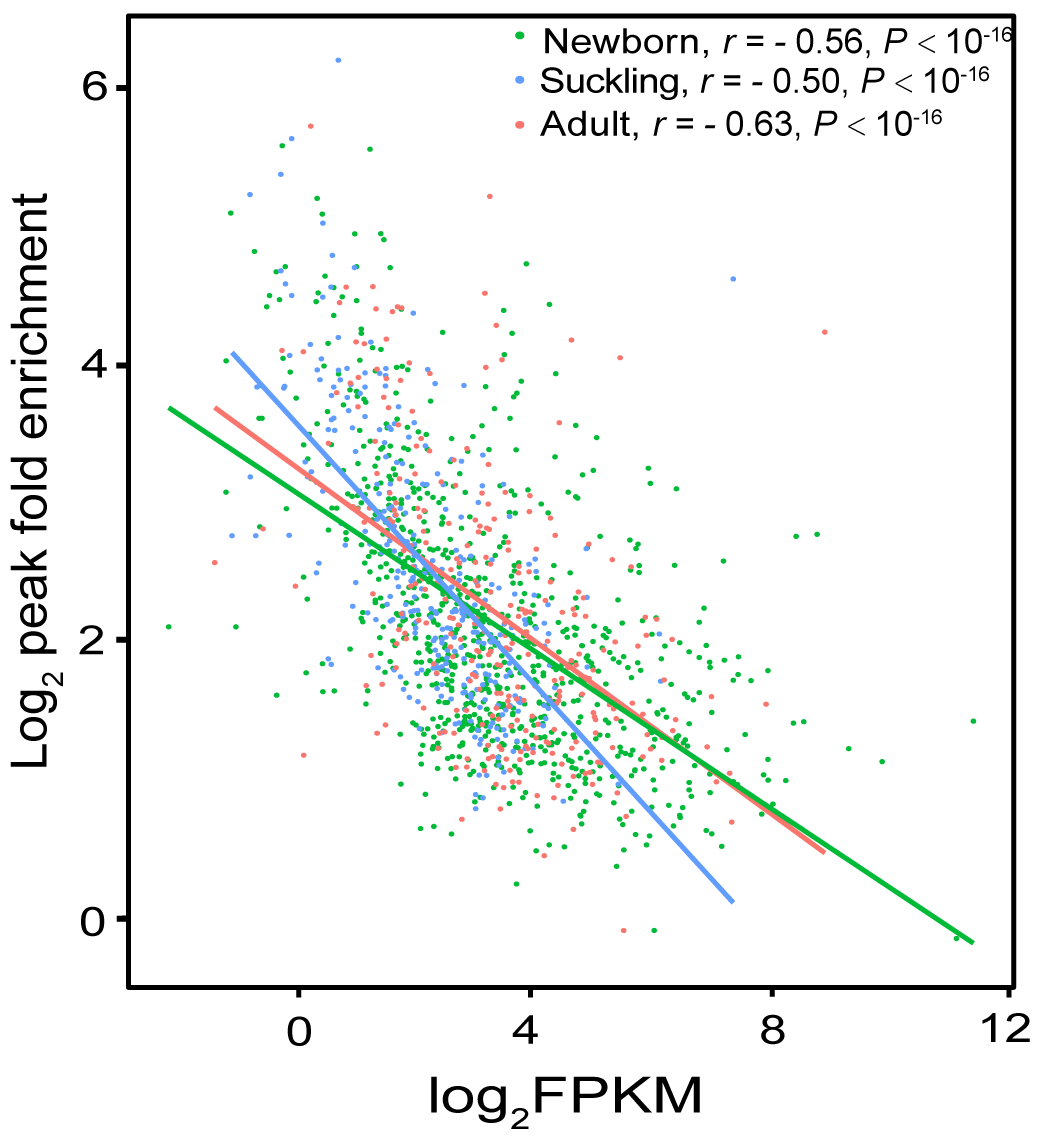

Supplement: S5 Fig — A higher negative correlation rate was found in all three stages (average Pearson’s r = -0.56, P < 10−16). (TIF) [file pone.0173421.s005.tif]
