# Supplementary material for: mRNA N6-methyladenosine methylation of postnatal liver development in pig
Source: PLoS One. 2017 Mar 7;12(3):e0173421. doi: 10.1371/journal.pone.0173421 (PMC5340393; doi:10.1371/journal.pone.0173421)
Supplement: S1 Table — (DOCX) [file pone.0173421.s006.docx]

**S1 Table**. Summary of sequenced and mapped data of the MeRIP-Seq and input RNA-seq samples.

|  | **Age** | **Individual** | **High quality data (Gb)** | **High quality data (exclude rRNA) (Gb)** | **Mapped ratio (%)** | **Uniquely mapped ratio (%)** | **Number of m^6^A narrow peaks \| peaks in intragenic region** | **m^6^A modified genes** | **Expressed**  **Genes (FPKM > 0.1)** |
| --- | --- | --- | --- | --- | --- | --- | --- | --- | --- |
| **MeRIP** | 0 day | Newborn 1 | 12.65 | 10.02 | 87.04 | 75.66 | 11,101 \| 8,984 | 4,872 | \ |
|  |  | Newborn 2 | 11.06 | 8.55 | 87.87 | 76.13 | 10,778 \| 8,685 | 4,866 | \ |
|  |  | Newborn 3 | 12.03 | 9.39 | 89.81 | 77.95 | 10,782 \| 8,836 | 4,933 | \ |
|  | 21 days | Suckling 1 | 10.38 | 8.74 | 89.27 | 82.16 | 9,164 \| 7,366 | 4,433 | \ |
|  |  | Suckling 2 | 9.89 | 8.32 | 88.47 | 83.82 | 7,125 \| 5,883 | 3,153 | \ |
|  |  | Suckling 3 | 12.38 | 10.18 | 88.72 | 81.59 | 9,632 \| 7,801 | 4,532 | \ |
|  | 2 years | Adult 1 | 12.80 | 10.18 | 89.83 | 84.04 | 10,231 \| 8,132 | 4,635 | \ |
|  |  | Adult 2 | 10.61 | 8.30 | 90.44 | 83.14 | 9,907 \| 7,953 | 4,616 | \ |
|  |  | Adult 3 | 12.83 | 10.28 | 88.63 | 83.12 | 8,710 \| 7,039 | 4,226 | \ |
| **Input** | 0 day | Newborn 1 | 13.00 | 9.73 | 90.01 | 78.52 | \ | \ | 13,527 |
|  |  | Newborn 2 | 10.30 | 7.85 | 90.38 | 79.36 | \ | \ | 13,780 |
|  |  | Newborn 3 | 10.17 | 7.77 | 91.04 | 79.46 | \ | \ | 13,536 |
|  | 21 days | Suckling 1 | 8.74 | 6.72 | 91.50 | 86.75 | \ | \ | 13,601 |
|  |  | Suckling 2 | 10.90 | 8.97 | 90.07 | 86.80 | \ | \ | 13,613 |
|  |  | Suckling 3 | 9.51 | 7.20 | 89.44 | 86.71 | \ | \ | 13,638 |
|  | 2 years | Adult 1 | 10.41 | 7.73 | 94.01 | 86.47 | \ | \ | 13,532 |
|  |  | Adult 2 | 9.27 | 7.03 | 93.44 | 85.93 | \ | \ | 13,484 |
|  |  | Adult 3 | 8.02 | 6.00 | 92.16 | 86.56 | \ | \ | 13,489 |

Narrow peaks: original peaks called by MACS2 software.
