# Supplementary material for: mRNA N6-methyladenosine methylation of postnatal liver development in pig
Source: PLoS One. 2017 Mar 7;12(3):e0173421. doi: 10.1371/journal.pone.0173421 (PMC5340393; doi:10.1371/journal.pone.0173421)
Supplement: S2 Table — (DOCX) [file pone.0173421.s007.docx]

**S2 Table**. Number of expressed genes, merged peaks and proportion of m^6^A modified transcripts in each group.

| **Group** | **Number of expressed genes (FPKM> 0.1)** | **m^6^A modified genes** | **Number of m^6^A peaks** | **m^6^A peaks in intragenic regions** | **Number of m^6^A peaks per modified gene** |
| --- | --- | --- | --- | --- | --- |
| Newborn | 13,326 | 4,676 (35.09%) | 8,855 | 6,626 (74.83%) | 1.42 |
| Suckling | 13,495 | 4,103 (30.40%) | 7,350 | 5,453 (74.19%) | 1.33 |
| Adult | 13,307 | 4,339 (32.61%) | 7,961 | 5,868 (73.71%) | 1.35 |
