# Supplementary material for: mRNA N6-methyladenosine methylation of postnatal liver development in pig
Source: PLoS One. 2017 Mar 7;12(3):e0173421. doi: 10.1371/journal.pone.0173421 (PMC5340393; doi:10.1371/journal.pone.0173421)
Supplement: S5 Table — (DOCX) [file pone.0173421.s010.docx]

**S5 Table**. Diverse patterns of m^6^A motif sequences (RRm^6^ACH).

| **Motif sequences** | **Number of summits with motif** | **Number of summits without motif** | | **Percentage (%)** | |
| --- | --- | --- | --- | --- | --- |
| GGACC | 15,420 | 54,716 | 21.99 | |  |
| GGACT | 15,288 | 54,848 | 21.80 | |  |
| GGACA | 14,258 | 55,878 | 20.33 | |  |
| AGACT | 10,309 | 59,827 | 14.70 | |  |
| AGACC | 8,197 | 61,939 | 11.69 | |  |
| GAACT | 8,187 | 61,949 | 11.67 | |  |
| AGACA | 7,730 | 62,406 | 11.02 | |  |
| GAACA | 6,227 | 63,909 | 8.88 | |  |
| GAACC | 6,091 | 64,045 | 8.68 | |  |
| AAACT | 5,343 | 64,793 | 7.62 | |  |
| AAACA | 5,040 | 65,096 | 7.19 | |  |
| AAACC | 4,454 | 65,682 | 6.35 | |  |
